# Case report of neuronopathic mucopolysaccharidosis type II: Early intracerebroventricular enzyme replacement therapy and hematopoietic cell transplantation with developmental outcomes up to 5 years of age

**DOI:** 10.1016/j.ymgmr.2025.101254

**Published:** 2025-09-25

**Authors:** Azuma Ikari, Asahito Hama, Torayuki Okuyama

**Affiliations:** a Department of Pediatrics, Anjo Kosei Hospital, 28 Higashihirokute, Anjo-cho, Anjo, Aichi 446-8602, Japan; b Department of Hematology and Oncology, Children's Medical Center, Japanese Red Cross Aichi Medical Center Nagoya First Hospital, 3- 35 Michishita-cho, Nakamura-ku, Nagoya 453-8511, Japan; c Department of Clinical Genomics, Saitama Medical University, 1397-1 Yamane, Hidaka, Saitama 350-1241, Japan

**Keywords:** Neuronopathic MPSII, HCT, Intracerebroventricular administration, Missense variant, Residual enzymatic activity, QOL

## Abstract

Neuronopathic mucopolysaccharidosis type II (MPS II) is a severe lysosomal storage disorder associated with early-onset developmental regression and a poor prognosis. Although enzyme replacement therapy (ERT) and hematopoietic cell transplantation (HCT) have been employed to address systemic symptoms, they have not demonstrated sufficient efficacy in treating central nervous system (CNS) involvement. The intracerebroventricular administration of idursulfase beta has emerged as a novel approach for targeting CNS manifestations. Here, we report the clinical course of a male patient diagnosed with neuronopathic MPS II at 2 months of age, based on family history and genetic analysis. Intravenous ERT was initiated early, followed by the introduction of intracerebroventricular idursulfase beta at 10 months, and HCT at 23 months. Since then, only intracerebroventricular ERT has been continued. At 5 years of age, the patient exhibited age-appropriate neurodevelopment, stable cognitive function, and normal physical growth without signs of developmental regression. Imaging findings remained stable, and cerebrospinal fluid biomarkers normalized. Notably, the patient harbored a missense variant potentially associated with residual enzymatic activity, which may have contributed to favorable outcomes. To our knowledge, this is the first reported case in which normal development was maintained until 5 years of age in a patient with neuronopathic MPS II. This case highlights the potential benefits of early diagnosis and a multimodal therapeutic strategy, including intracerebroventricular ERT and HCT, for preserving neurodevelopment. It also holds significance as a rare but valuable example, suggesting the efficacy of a novel treatment paradigm for a condition traditionally associated with a poor prognosis.

## 1 Introduction

Mucopolysaccharidosis type II (MPS II; Hunter syndrome) is a disorder caused by a deficiency in iduronate-2-sulfatase (IDS), which leads to the accumulation of glycosaminoglycans (GAGs), specifically dermatan sulfate and heparan sulfate. Accumulation of GAGs results in a wide spectrum of clinical manifestations. The disease is characterized by systemic symptoms, such as short stature, joint contractures, valvular heart disease, hepatosplenomegaly, skeletal deformities, and sleep apnea. Approximately 70 % of the patients exhibit central nervous system (CNS) involvement, including developmental delays and regression. These cases are classified as the neuronopathic type—distinguished from the attenuated type—which does not present with CNS symptoms.

Hematopoietic cell transplantation (HCT) has long been used as a treatment option for this disorder [1]. In 2007, the first enzyme replacement therapy (ERT), intravenous idursulfase (elaprase), was approved [2]. ERT has shown benefits such as increased six-minute walk distance, improved respiratory function, and reduced hepatosplenomegaly; however, its efficacy against CNS symptoms has not been demonstrated [3]. Although HCT improves somatic manifestations and may provide greater benefit than ERT for CNS symptoms, its efficacy remains limited and is insufficient to preserve normal neurodevelopment [1]. Severe adverse events, such as graft-versus-host disease (GVHD) and transplant-related mortality, have also been reported [1].

In 2021, two new therapeutic agents with potential efficacy in the CNS became available. Idursulfase beta (Huntrarase®) is administered directly into the cerebral ventricles and requires the prior placement of a cerebrospinal fluid (CSF) reservoir. Once in place, the drug can be administered monthly in just a few minutes [4,5]. Pabinafusp alfa (Izcargo®) is a modified enzyme designed to cross the blood-brain barrier (BBB); although it requires weekly intravenous infusions, it is also expected to exert therapeutic effects on the CNS [6,7].

During the natural course of severe MPS II, CNS symptoms typically progress as follows: motor development is relatively normal until approximately 1 year of age, but language delay becomes apparent thereafter. By approximately 2–3 years of age, developmental regression begins, with progressive motor dysfunction becoming increasingly evident, and most patients becoming bedridden by the age of 10 [3]. As symptom progression is thought to be associated with the accumulation of GAGs within the brain parenchyma, initiating CNS-targeted interventions as early as possible may contribute to improved outcomes.

Here, we report a favorable developmental course up to the age of 5 years in a patient who was diagnosed with neuronopathic MPS II at 2 months of age based on family history and genetic analysis. The patient initially received intravenous idursulfase and intracerebroventricular administration of idursulfase beta was initiated at 10 months of age. Subsequently, HCT was performed at 1 and 11 months of age. Since then, the patient has continued to receive only intracerebroventricular idursulfase beta. To the best of our knowledge, this is the first reported case of a patient with neuronopathic MPS II showing favorable neurodevelopmental outcomes up to 5 years of age, suggesting the potential benefit of early initiation of intracerebroventricular idursulfase beta.

## 2 Case report

The patient was a 5-year-old boy at the time of writing the manuscript. His family history was notable for a maternal uncle diagnosed with severe MPS II who died at the age of 13 years. During the prenatal period, the mother received routine care at a local obstetric clinic and the pregnancy was uneventful. At 38 weeks and 1 day of gestation, the patient was delivered via cesarean section because of a breech presentation. At birth, his weight was 2856 g (+0.1 SD), length was 45.5 cm (−1.3 SD), and head circumference was 35.8 cm (+2.1 SD). The Apgar scores were 9 at 1 min and 10 at 5 min.

After birth, the patient experienced respiratory distress, including tachypnea and chest retraction, and required 30 % oxygen supplementation to maintain adequate oxygen saturation. He was transferred to the neonatal intensive care unit (NICU) of our hospital, where he was diagnosed with transient tachypnea of the newborn and managed with oxygen therapy and continuous positive airway pressure (CPAP). Physical examination on admission revealed extensive Mongolian spots across the trunk and extremities. No dysmorphic facial features, abnormal muscle tone, or external malformations were observed. Laboratory tests revealed no abnormalities. Chest and abdominal radiographs revealed no evidence of cardiomegaly or skeletal abnormalities. Echocardiography revealed a ventricular septal defect (VSD) measuring approximately 2 mm in the muscular portion of the septum; however, no valvular disease or myocardial hypertrophy was observed. The patient's clinical course during hospitalization was also favorable. CPAP was discontinued on day 3 of life, and the patient demonstrated good oral feeding and weight gain. The patient was discharged on day 6 of life.

During hospitalization, MPS II was considered based on the family history and physical findings, prompting further evaluation. Urinary GAG analysis revealed an elevated total uronic acid level of 355 mg/g creatinine, with fractionation analysis showing dermatan sulfate 53 %, heparan sulfate 8 %, and chondroitin sulfate 39 %. Leukocyte enzyme assay demonstrated IDS activity below the quantification limit (<1.0 nmol/mg/4 h), confirming the diagnosis. Genetic testing identified the patient as hemizygous for a previously reported missense variant of IDS (exon 3 c.257C > G/p. P86R). This variant has been detected in multiple patients with neuronopathic MPS II. The patient's maternal uncle, who likely harbored the same variant, died at 13 years of age. Based on this genetic background and family history, CNS involvement was considered likely [8].

**Fig. 1:**
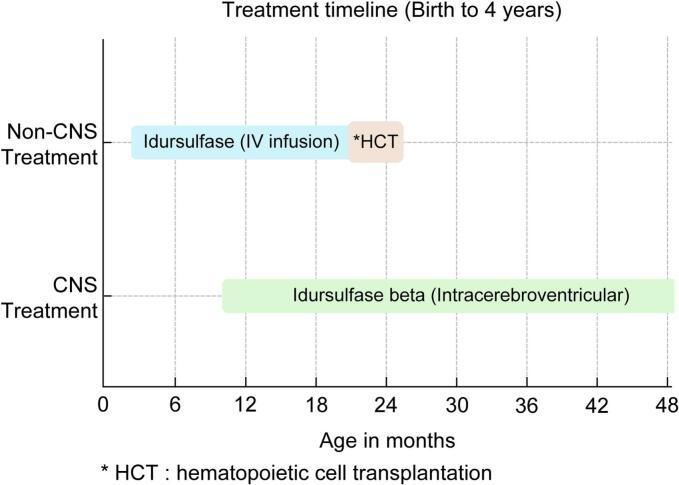
**Treatment course.** Intravenous infusion of idursulfase at 0.5 mg/kg was administered over 1–3 h until 23 months of age, at which point hematopoietic cell transplantation (HCT) was performed and idursulfase was discontinued. At 10 months of age, a cerebrospinal fluid (CSF) reservoir was implanted; since then, intraventricular administration of idursulfase beta at a dose of 30 mg has been performed every 4 weeks, with each infusion completed in approximately 1 min.

The treatment course is summarized in Fig. 1. From 2 months of age, the patient received weekly intravenous idursulfase (0.5 mg/kg over 1–3 h). After undergoing HCT at 23 months of age, idursulfase was discontinued. At 10 months, a CSF reservoir was implanted and intracerebroventricular idursulfase beta (30 mg) was administered every 4 weeks in an outpatient setting, with each administration completed in approximately 1 min. This regimen was maintained during hospitalization for HCT.

**Fig. 2:**
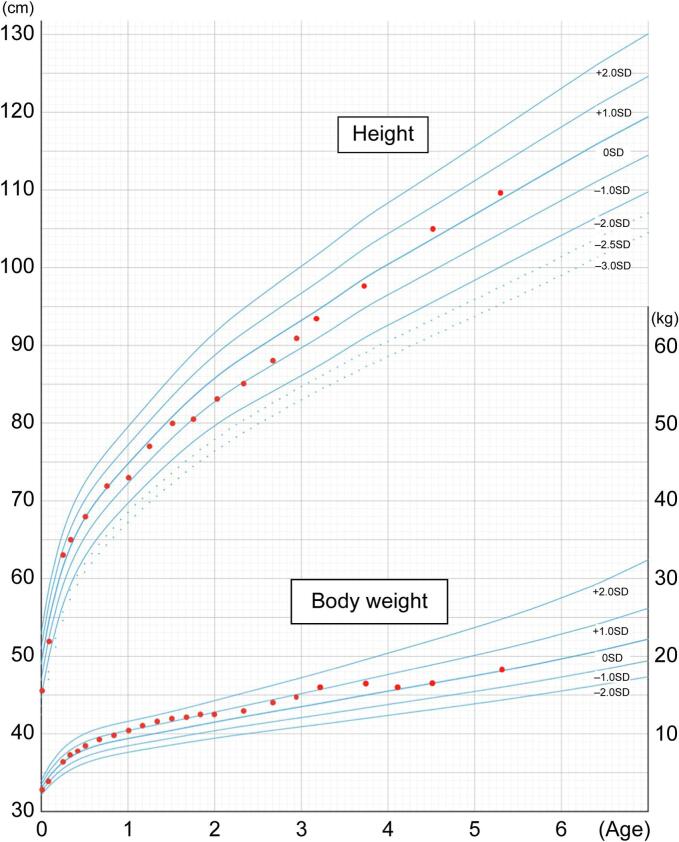
**Growth trajectory of body weight and height.** Both parameters remained within normal ranges throughout the postnatal period.

At 9 months, urinary uronic acid levels had decreased to 40.6 mg/g creatinine, within the normal range. Periodic evaluations including abdominal ultrasonography, echocardiography, skeletal radiography, electrocardiography, blood tests, and urinalysis revealed no significant abnormalities. Ophthalmologic, otolaryngologic, and oral surgical examinations were unremarkable, and follow-up was discontinued by the age of 3 years. The bilateral inguinal and umbilical hernias were surgically treated at 2 years of age. Growth parameters remained within normal ranges for up to 5 years, with no evidence of short stature (Fig. 2).

**Fig. 3:**
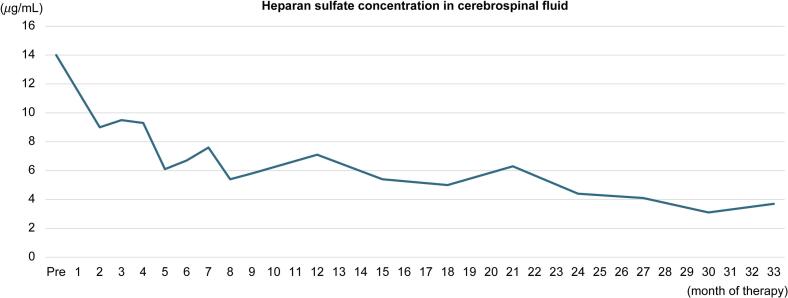
**Longitudinal changes in heparan sulfate concentration in cerebrospinal fluid after initiation of therapy.** The concentration was elevated at 14.0 µg/mL before treatment, but showed a decreasing trend following the initiation of therapy and eventually stabilized within the range of 3–5 µg/mL.

**Fig. 4:**
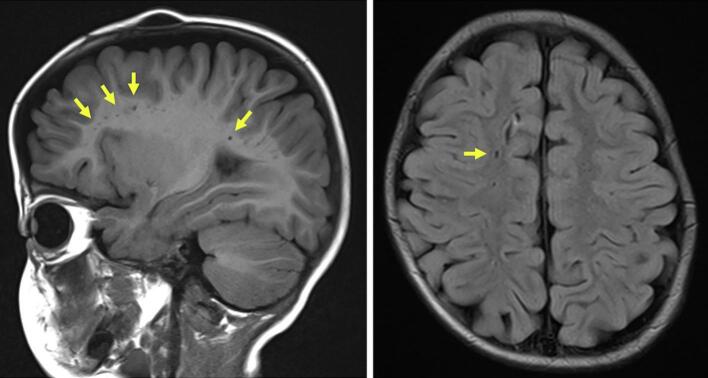
**Cranial MRI findings (performed annually).** At 1 year of age, multiple scattered enlarged perivascular spaces were observed, giving a vacuolated appearance (arrows). No progression of these findings has been observed on subsequent annual follow-up imaging. No ventricular enlargement was noted.

**Fig. 5:**
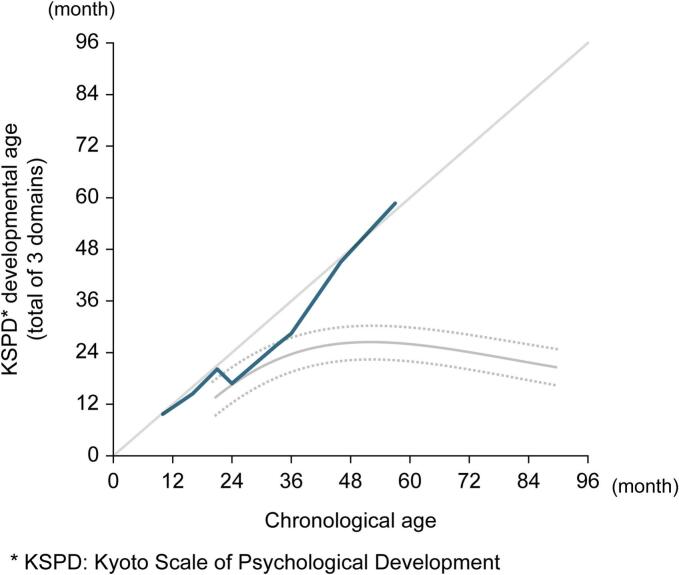
**Chronological changes in developmental age.** Although motor development was favorable, a delay in language development was observed. By approximately 2 years of age, a noticeable gap between language developmental age and chronological age was evident. However, from approximately 3 years of age, language development accelerated, reaching age-appropriate levels by around 4 years of age and has since remained stable. The solid and dashed lines in the graph represent the mean developmental trajectory (solid line) and the 95% confidence interval (dashed line) of the control group receiving weekly intravenous infusions of idursulfase.

Regarding the CNS, CSF heparan sulfate was elevated at 14.0 μg/mL prior to therapy but has remained at 3–5 μg/mL since treatment initiation (Fig. 3). Cranial MRI at 1 year showed multiple scattered, enlarged perivascular spaces; however, annual follow-up imaging showed no progression (Fig. 4). The developmental milestones are summarized in Table 1. Motor and cognitive development were within normal limits at an early stage. Although language development was delayed, with only one meaningful word at age two, vocabulary rapidly increased from 2.5 years, and by the age of 3 years, the patient could form three-word sentences. Developmental testing was conducted once or twice a year using the Kyoto Scale of Psychological Development 2001. At the age of 2 years, the developmental quotient (DQ) was slightly reduced due to delayed language development, but after the age of 3 years, it remained at approximately 100 (Fig. 5). At the age of 5 years, the patient exhibited no signs of neurodevelopmental disorders, and development remained favorable. Monthly intracerebroventricular administration of idursulfase beta in the outpatient setting is ongoing and will be continued.

**Table 1:** **Developmental milestones.** Motor development was favorable, although the acquisition of meaningful words was delayed. After the emergence of words, language development progressed rapidly.

Developmental milestone	Age of acquisition (Months)
Head control	3
Sitting without support	6
Pulling to stand	8
Cruising	10
Standing independently	11
Walking	13
Babbling	8
Using 1 meaningful word	24
Using 2 meaningful words	28
Using 5 or more meaningful words	30
Using 2-word phrases	33
Using 3-word phrases	35

## 3 Discussion

The patient was diagnosed with a severe form of MPS II, and ERT was initiated in the early neonatal period. After 10 months, intracerebroventricular idursulfase beta therapy was initiated. HCT was performed at the age of 2 years; since then, the patient has been managed solely with monthly intracerebroventricular ERT. The patient showed favorable developmental and growth outcomes and an improved quality of life (QOL). This case suggests that in addition to early diagnosis, a multimodal therapeutic approach, including intracerebroventricular ERT and HCT, may help to control disease progression and improve prognosis.

Metabolic disorders, including MPS, present with a wide range of clinical manifestations owing to the accumulation of undegraded substrates. Therefore, early diagnosis and treatment initiation before significant substrate accumulation may help to slow disease progression and maintain health. This also applies to the CNS, where early intervention is necessary before irreversible changes occur. In this case, the diagnosis was prompted by the family history; however, familial MPS II is rare. Thus, the implementation of newborn screening is essential for early diagnosis.

MPS II shows a relatively clear genotype–phenotype correlation. In our previous study involving 65 Japanese families with MPS II, nearly all patients with the attenuated-type carried missense variants. In contrast, most patients with the neuronopathic type harbor variants that are unlikely to retain residual enzyme activity, such as large deletions, recombination with the IDS2 pseudogene, nonsense variants, or splice-site mutations [8]. Furthermore, neurodegeneration progresses more gradually in patients with neuronopathic MPS II with missense variants than in patients with other types of mutations [3].

In this case, neurodevelopmental outcomes remained favorable. Contributing factors include the presence of a missense variant, early treatment initiation, and early HCT. These factors may have enhanced the efficacy of intracerebroventricular ERT, and the marked improvement in language development observed after the age of 2 is also considered to be the result of these combined factors. Recent studies have similarly demonstrated that intracerebroventricular administration of idursulfase beta can reduce CSF heparan sulfate levels and help to maintain developmental age in patients with neuronopathic MPS II.

Although HCT has historically been avoided owing to its risks and limited CNS efficacy, advances in transplantation have reduced complications, prompting reevaluation [9]. Weekly systemic ERT can significantly reduce the QOL. In contrast, the combined approach used here, monthly intracerebroventricular ERT with HCT, can be managed through brief outpatient procedures, and substantial improvement in QOL can be expected. Although HCT still poses risks, its combination with intracerebroventricular ERT offers a promising strategy for treating CNS symptoms and may support renewed consideration of HCT as a viable treatment option. It should be noted that we did not perform a quantitative assessment using standardized QOL scales in this report. Objectively demonstrating QOL improvement is an important aspect of evaluating treatments, and it may also serve as a critical indicator for families when making treatment decisions; therefore, the use of QOL scoring should be considered in the future.

Neuronopathic MPS II is a severe disorder with a poor prognosis. To date, no cases have been reported in which normal development has been continuously maintained for up to five years. However, this case demonstrates that sustained normal development may be achievable through early diagnosis, family history, or newborn screening, and an appropriate combination of therapies, including intravenous ERT, HCT, and intracerebroventricular enzyme administration.

This patient harbored a missense variant, potentially allowing for some residual enzyme activity, which may have contributed to the favorable outcome. A limitation of this study is that we cannot determine whether similar outcomes can be achieved in patients with variants that lack residual enzyme activity. Future research should investigate this question through clinical trials that include patients with various genetic mutations. Additionally, longer follow-up periods beyond 5 years would be valuable to assess the durability of treatment effects.

## 4 Conclusion

This case report highlights the potential benefits of early diagnosis and a multimodal therapeutic approach for patients with neuronopathic MPS II. The combination of intracerebroventricular ERT and HCT resulted in favorable neurodevelopmental outcomes up to 5 years of age, suggesting that this approach may help to preserve cognitive function in these patients. Our findings support the importance of newborn screening for early diagnosis and intervention before significant accumulation of GAGs and irreversible CNS damage occur. The genotype-phenotype correlation in MPS II suggests that patients with missense variants may have a better response to treatment, though further studies are needed to confirm this observation. This case provides valuable insights for clinicians managing patients with this devastating disorder and offers hope for improved outcomes through early, targeted interventions.

## CRediT authorship contribution statement

**Azuma Ikari:** Writing – review & editing, Writing – original draft, Visualization, Validation, Resources, Investigation, Data curation, Conceptualization. **Asahito Hama:** Validation, Resources, Investigation. **Torayuki Okuyama:** Writing – review & editing, Validation, Supervision, Resources, Methodology, Conceptualization.

## Consent for publication

Written informed consent for publication of the clinical details and images in this case report was obtained from the patient's guardian.

## Ethical approval

This study was conducted in accordance with the Declaration of Helsinki and approved by the institutional review board of Anjo Kosei Hospital (approval number: C25–008, date: 2025/5/2). Written informed consent was obtained from the patient's guardian.

## Declaration of generative AI and AI-assisted technologies in the writing process

During the preparation of this study, ChatGPT (OpenAI) was used to improve language clarity. After using this tool, the author reviewed and edited the content as required and took full responsibility for the content of the publication.

## Funding

This research is partly supported by Research on Rare and Intractable Diseases (Grant Number 23FC1032), awarded to Torayuki Okuyama under the Health, Labour and Welfare Sciences Research Grants. The title of the grant is Research on establishing a medical care system that enables early diagnosis and treatment of lysosomal diseases and peroxisomal diseases (including adrenoleukodystrophy).

## Declaration of competing interest

The authors declare that they have no known competing financial interests or personal relationships that could have appeared to influence the work reported in this paper.

## Data Availability

No data was used for the research described in the article.
